# Study on Geometry, Dimensional Accuracy and Structure of Parts Produced by Multi Jet Fusion

**DOI:** 10.3390/ma14164510

**Published:** 2021-08-11

**Authors:** Martyna Adach, Paweł Sokołowski, Tomasz Piwowarczyk, Krzysztof Nowak

**Affiliations:** 1 3D Center Sp. z o.o., Kwiatkowskiego 4, 52-407 Wroclaw, Poland; martyna.adach@3dcenterpolska.pl (M.A.); krzysztof.nowak@3dcent.com (K.N.); 2 Department of Metal Forming, Welding and Metrology, Faculty of Mechanical Engineering, Wroclaw University of Science and Technology, Wyb. Wyspianskiego 27, 50-370 Wroclaw, Poland; tomasz.piwowarczyk@pwr.edu.pl

**Keywords:** additive manufacturing, multi jet fusion, build orientation, geometrical accuracy, microstructure

## Abstract

Multi Jet Fusion (MJF) is one of the newest additive manufacturing technologies for polymer powders, introduced in recent years. This fully industrial technology is gaining big interest as it allows fast, layer-by-layer, printing process, short production cycle, and very high printing resolution. In this paper, twelve thin-walled, spherical PA12 prints were studied in terms of geometry, dimensional accuracy, and fracture surface characteristics. The various characteristic features for MJF prints were observed here for parts produced according to four various print orientations and having different thicknesses, i.e., 1, 2 or 3 mm. The study showed that MJF technology can print such difficult shapes. However, the set of parameters allowing producing parts with highest geometrical and dimensional accuracy causes at the same time some microstructural issues, like great interlayer porosity or high number of non-processed powder particles embedded in the print structure.

## 1. Introduction

Additive manufacturing (AM) is one of the pillars of a new industrial era called Industry 4.0. It brings together new solutions for the development of intelligent production and technology automation. A new approach to industry includes, in addition to AM, the Internet of Things, Industrial Internet, Smart Manufacturing and Cloud-based Manufacturing [1].

The first 3D printing technology, called stereolithography (SLA), was invented in the 1980s by Charles Hull and consists of resin hardened using UV light. Then, in 1986, Charles Hull founded the first 3D printing company, 3D Systems [2]. The idea of additive manufacturing, unlike conventional, subtractive manufacturing methods, considers the creation of parts layer by layer. Additive manufacturing enables production of a physical object directly from a digital model, done usually by 3D modeling, 3D scanning or computed tomography. Thanks to AM, it is possible to create a product in any place and at any time just by transferring and processing the CAD file.

One of the most important advantages of AM is the possibility of manufacturing customized products [3]. The product customization for specific customers’ demands is easier and cheaper in comparison to conventional manufacturing technologies [4]. Additive manufacturing can easily handle complicated geometry, joining of a few parts into one, making cooperating elements, etc. However, the design freedom has obviously some limitations. There is no specific guidance on model development. Most engineers must acquire this knowledge through experiments and further experience. It is also worth mentioning that, despite the significant development in AM in recent years, the number of professional instructions and international standards is still very limited [5].

In the context of AM, the scientific literature mainly addresses new applications areas, novel materials, and optimization of 3D printing processes for obtaining the highest quality of prints [6]. Medicine is one of the fields where AM is used increasingly. Bone tissue engineering, like scaffolds [7], implants and anatomical models are some of the common applications [8]. Other uses of AM usually include aerospace, automotive, architecture and art [9,10]. Material development for AM mainly focuses on their strength properties. New alloys are investigated and developed rather than pure metals. For example, Tawlik et al. studied enhanced properties of aluminum alloys [11], Wanga et al. examined nickel-based superalloy with high chromium content [12] and Peng et al. produced and studied CoCrFeNiMn cobalt-based alloy [13]. The research on polymers includes mainly thermoplastic ones such as ABS (acrylonitrile bitadiene styrene), PLA (polylactide), PA (polyamide) or PC (polycarbonate), produced in various forms depending on the printing technology [14,15].

According to ISO/ASTM 52900:2015 [16] standard, additive technologies could be divided into seven process categories: vat photopolymerization, material extrusion, powder bed fusion, material jetting, binder jetting, sheet lamination and direct energy deposition. Power bed fusion (PBF) processes are usually used in industry because they enable the production of many elements at once, including quite large structures thanks to relatively big working platforms [15]. In this work, prints made by Multi Jet Fusion, which belongs to PBF technology, are examined, and therefore this technology is briefly discussed below.

**Figure 1:**
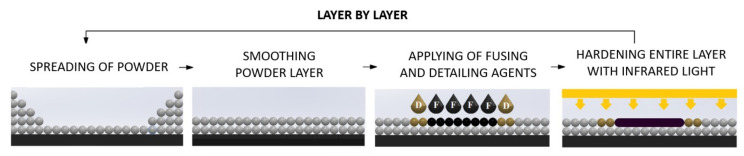
Multi Jet Fusion printing scheme.

Hewlett-Packard introduced their own 3D printer working in a patented technology called Multi Jet Fusion (MJF) in 2014. The MJF printing process begins with applying a layer of powder on the top of build platform using a roller. The thickness of each layer is around 80 microns. Then, fusing and detailing agents are used and the whole layer is exposed to an infrared light. Fusing agent determines which places in the layer are bonded and detailing agent needs to endure sharp and smooth edges by limiting the bonding of powder near to the print surface. The following stages in MJF process are shown in Figure 1.

The access to certified materials dedicated to MJF is still limited. Nowadays it is possible to use the following materials: polyamide 12 (PA12), polyamide 11 (PA11), polyamide 12 reinforced with glass beads (PA12GB), polypropylene (PP) and thermoplastic polyurethane elastomer (TPU). The average size of powders ranges from 54–77 µm [17].

One of the important topics in the field of additive manufacturing is the characterization of geometrical/dimensional accuracy and structure of prints. The main reason is too low quality of the prints, which is influenced by the process parameters and the orientation of prints in the working platform. On the one hand, to improve the quality of printed elements, it is necessary to understand the impact of different process parameters on the final print’s accuracy [18]. On the other hand, there is a big interest in rapid verification of 3D prints made in various AM technologies. The quick control of prints is often aimed at selecting the most optimal printing parameters or comparing the quality of own prints with others, including the benchmarks. Usually, the preliminary 3D print geometry and structure analyses can be done by [19]:

- Measurements of the print using measuring tools such as calipers and micrometric screws;
- 3D scanning and digital measurements or analyses;
- Coordinate measuring machines (CMMs);
- Observations under light or scanning electron microscope;
- Computed tomography (usually X-ray microtomography).

The considerations on how to truly measure the geometric accuracy of 3D prints are important issues nowadays. The most popular method is 3D scanning [20,21,22,23,24], which allows to easily compare the resulting print’s 3D scan with the 3D model. For the purpose of 3D reconstruction, Geomegic Control or GOM Inspect software is widely applied. Then, the different approaches for comparing scan geometry with CAD design, including: volumetric 4-points congruent sets (4PCS) algorithm, selecting point pairs and iterative nearest point algorithm (IPC) are used [25]. Another popular method is to use the CMM testing machine, studied e.g., in [23,26]. The other methods used for additive manufacturing, mentioned in the literature, are usually: photogrammetry [27,28], computed tomography [26], and usually for simple linear measurements, precision hand tools, like digital caliper [28] or micrometer screw [29]. In addition, [30] is a review in the field of dimensional and geometrical verification but also presents the tools for improving the accuracy of prints made in various 3D printing technologies. Newly used tools for process optimization include: Finite Element Method (FEM) analysis, parameter selection through the design of experiments (DoE), development of algorithms based on neural networks and parametric tests.

The geometrical and dimensional accuracy of prints is verified based on different authorial models, some of them are of simple geometry, and others are of very complicated shapes. However, the method used for measuring the print geometry depends mainly on the shape characteristics of the element. In addition, the dimensional deviations are connected with the size of the print and the manufacturing technology. One that is widely studied in this context is the Fused Deposition Modeling (FDM). The results of various studies show that the orientation of parts and the heat distribution in the process may strongly affect the geometrical and dimensional accuracy of prints [20,21,27,31]. For powder bed fusion processes, including SLS (Selective Laser Sintering), MJF (Multi Jet Fusion) and 3DP (Three-Dimensional Printing) [22,26,28,32,33]. The orientation in the print chamber is also considered as one of the main factors affecting the accuracy of parts together with the print volume. Similar findings were made for PolyJet technology (which is based on photopolymerizations) [20,23] and Direct Metal Laser Sintering (DMLS/SLM) working with metallic powders [29].

Curved elements are rather difficult for both, additive manufacturing technology and for geometrical/dimensional accuracy investigations, so they are not often present in the literature. Dziubek and Filip [34] proposed a solid part with a convex and concave spherical surface as a test model to verify the accuracy of the FDM technology. The study showed that convex spherical surfaces are usually printed with negative deviation, while for concave surface with a positive deviation both surfaces are staggered. It was also mentioned [35] that spherical surfaces are suitable standards for testing 3D imaging systems, which is additionally confirmed by ASTM (American Society for Testing and Materials) and NIST (National Institute of Standards and Technology).

The issues with printing spherical surfaces are related mainly to the specific layer thickness in each technology. The thicker the layer is, the greater the surface topography issues, i.e., well-known stair stepping effect. Moreover, the generation of .stl file for printing process can also be important. As this is a geometrical representation of the 3D model using triangular elements [36], an accurate mesh increases the dimensional accuracy of the prints itself. Otherwise, it causes the heterogeneous triangular structure of printed elements and significantly reduces the quality of the prints. However, the use of fine element size has an opposite effect on the processing and printing speed, so a compromise is necessary here.

According to the literature study, geometry, dimensional accuracy, and structure investigations are necessary to verify the quality of printed elements. Moreover, there is a lack of such research results for spherical elements printed in different orientations using powder bed fusion technologies. Therefore, the main aim of this work is to observe these characteristic features for spherical MJF prints produced according to four various print orientations and having different thicknesses. At the same time, it allows presenting the ability of MJF technology for producing highly curved objects.

Finally, in this work, twelve samples with different thickness (1, 2 or 3 mm) printed in four different orientation were examined. The linear scanner, digital microscopy, stylus profilometer and scanning electron microscopy were used to examine the geometry, dimensional accuracy, and structure of parts.

## 2. Materials and Methods

### 2.1. Parts Fabrication by Multi Jet Fusion

At first, spherical, thin-walled elements were designed and manufactured for geometry, dimensional accuracy, and structural investigations. All samples described below were manufactured by using HP Multi Jet Fusion 4200 3D printer (Hewlett-Packard, Barcelona, Spain) and processing station with fast cooling. The size of the printer’s chamber is 280 × 380 × 380 mm^3^. The spherical PA12 powder provided by HP with a mean diameter dv_50_ = 60 µm was used as a feedstock material. The other materials required in the MJF printing process were a fusing agent and detailing agent, both provided by HP as well.

**Figure 2:**
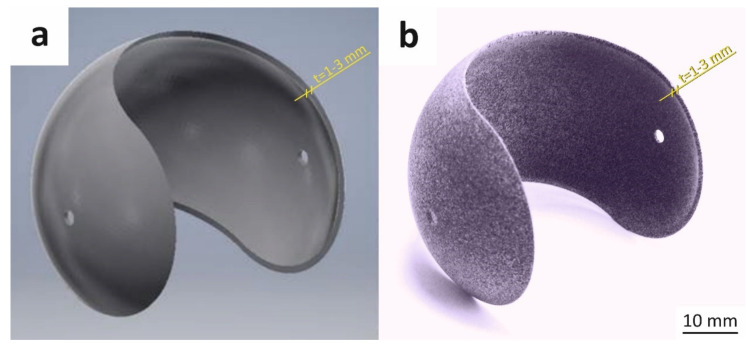
The overview of 3D model (**a**) and printed part (**b**).

**Figure 3:**
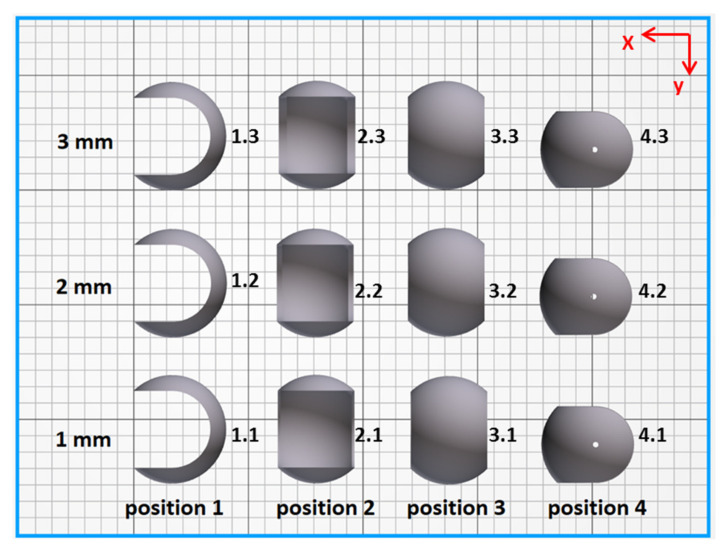
The orientation of analyzed parts in the printing chamber (top view); the samples are labeled X.Y, by position (X: 1–4) and thickness (Y: 1–3), respectively.

The model as well as the real part considered in this work are presented in Figure 2. Such spherical shape was chosen to emphasize differences in dimensional accuracy when changing the print thickness or its orientation in the printer chamber and to highlight the ability of MJF technology for producing such complex, highly curved objects. To study the influence of shell thickness on geometry, dimensional accuracy, and structure, three different versions of the initial model were prepared with 1 mm, 2 mm and 3 mm shell thickness. All other dimensions of the model were fixed, i.e., the external diameter of 60 mm and the diameter of the two holes equal to 3 mm. Furthermore, the influence of the build orientation is considered here and the four parts’ orientations in printing chamber are shown in Figure 3.

The printing chamber contained 12 spherical parts for this work and 40 other elements which corresponded in total to 6.39% packing density and 379.7 mm build height. The build unit was packed with a mixture of 20% new powder and 80% re-used powder, as recommended and usually applied in MJF. The balance mode was used for printing. This is one of four available modes and the most common set of parameters used in production by MJF. It ensures a good balance between mechanical properties and dimensional accuracy.

Once the printing process was completed, all parts were cooled down in the processing station before extraction. To ensure uniform cooling of all elements (up to room temperature), they were left in the build unit for 48 h. After that time, all elements were unpacked in the processing station. Excessive loose powder was removed with the use of HP post-processing station, for further re-use. The adhered powder was removed from the elements’ surfaces using a soft brush.

### 2.2. Roughness and Geometric Accuracy

All spherical samples were then scanned with a linear scanner RS3 integrated with Romer Absolute Arm 75201SI (Hexagon Manufacturing Intelligence, Montoire, France), for geometrical and dimensional accuracy investigations. The obtained scanned models were compared with relevant original .stl models using GOM Inspect software. The dimensions specified in the geometrical models were considered as a reference geometry. For each part, the resulting model-to-part deviations were recorded by GOM Inspect and provided for further analysis.

**Figure 4:**
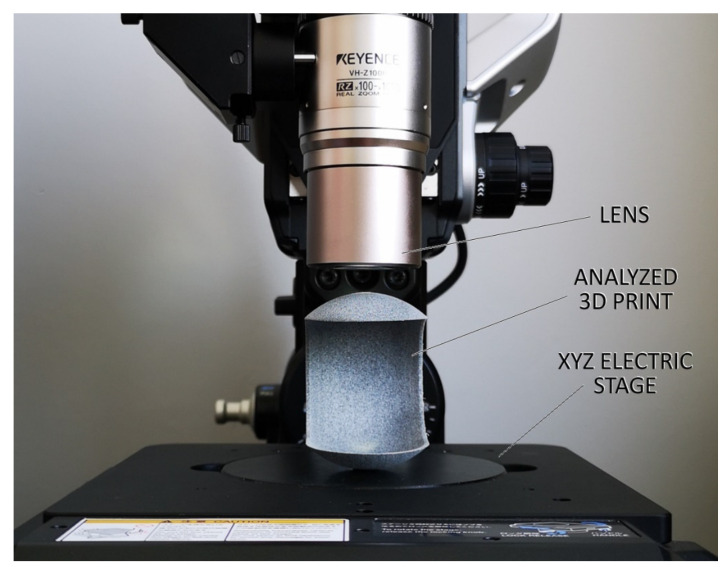
The setting of analyzed prints on the microscope for investigations.

The as-designed and as-printed parts were then analyzed in detail in one specific region, i.e., near to the hole introduced into the element. Such fine details are often difficult to be properly produced or introduced locally to some non-desired surface and/or microstructural effects. The observations and measurements were made using a Keyence VHX-6000 digital microscope (Keyence, Osaka, Japan). The setting of the examined elements under the microscope was the same each time (Figure 4).

**Figure 5:**
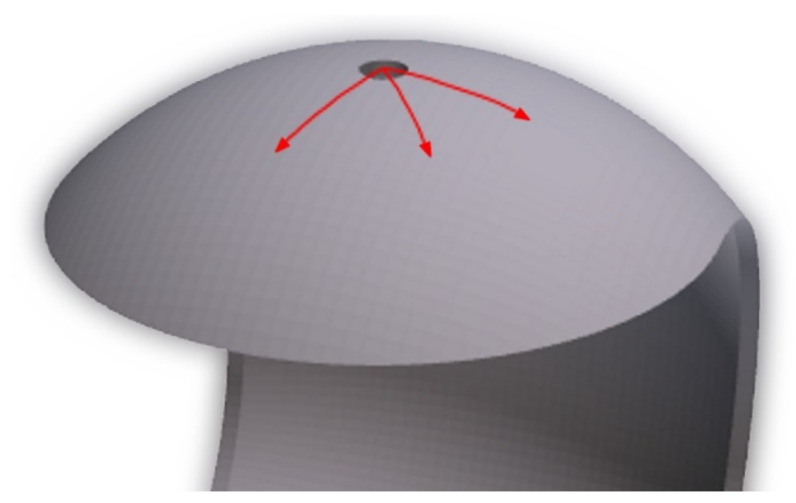
Scheme for roughness measurements, taken at the top, middle and bottom face of the print.

To accurately illustrate the measurable differences in quality of prints surface, roughness measurements were made by MarSurf PS 10 gauge (Mahr, Göttingen, Germany), in accordance with standard EN ISO 4288: 2011. The measurements were made over a distance of 4.8 mm and in the three directions, as shown in Figure 5. The selected roughness parameters, i.e., Ra, Rt and Rz were considered and compared.

### 2.3. Microstructure and Morphology

**Figure 6:**
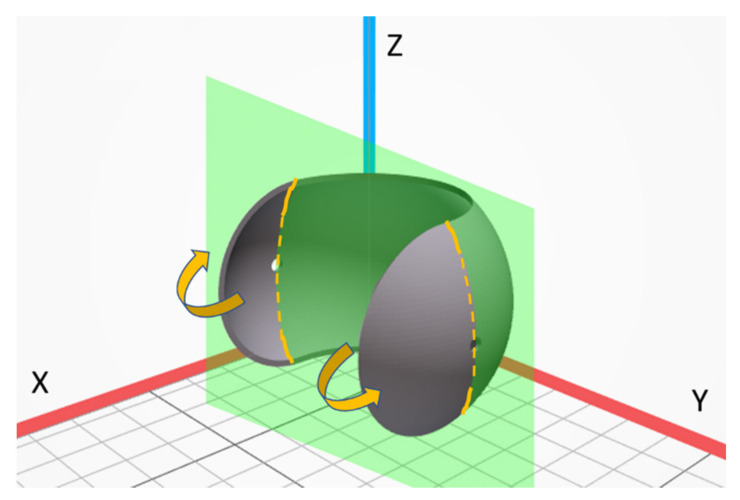
Fracture surface of printed parts for metallographic testing; green plane—fracture plane, arrows—direction of loading, continuous lines—pre-cracks, dashed lines—fracture surfaces for SEM studies.

The printed parts were partially cut and then sheared along the hole located on the side wall. This place was specified as the most difficult in the whole element structure. The way of loading, fracture plane and fracture surfaces are shown in Figure 6. The fracture surface orientation was always set similarly according to the studied element (not build orientation). The fracture surfaces of all elements were observed by Scanning Electron Microscope Tescan Vega 3 (TESCAN ORSAY HOLDING, Brno-Kohoutovice, Czech Republic). Prior the SEM investigations, the samples were gold-sputtered to avoid a charging effect. The scanning microscopy observations were intended at showing the microstructural features of prints with various thicknesses and produced under different build orientations. Typically, such analysis is applied to elements with simple geometry (e.g., tensile strength specimens), printed along the X, Y and Z axes. Here, much more complex elements were analyzed, and the observations were done for fractured surfaces. Furthermore, the surface area subjected to microstructural testing was formulated along the hole, which constitutes some structural notch in the whole structure. Such analysis should be more informative than for flat, ground, and polished samples, which should constitute some new understanding on the structure of MJF PA12 prints.

## 3. Results and Discussion

### 3.1. Geometric Accuracy

**Figure 7:**
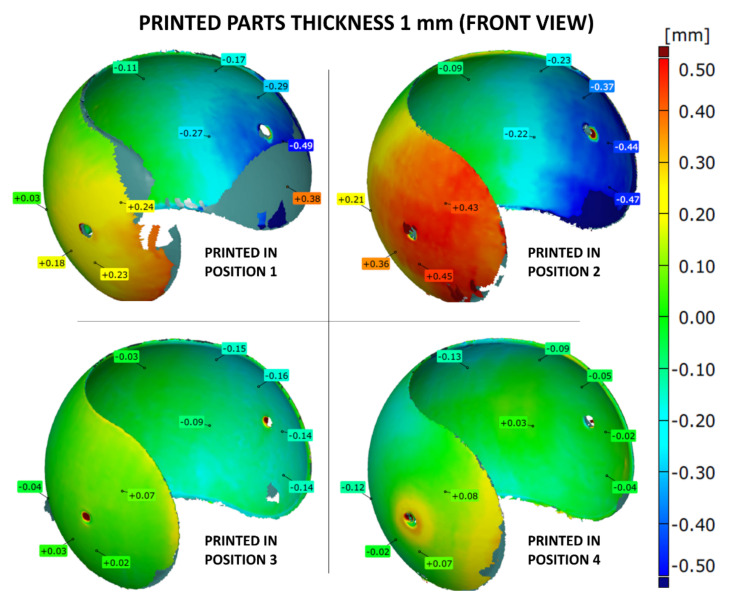
Geometry deviations of 3D printed part with a thickness of 1 mm (front view).

**Figure 8:**
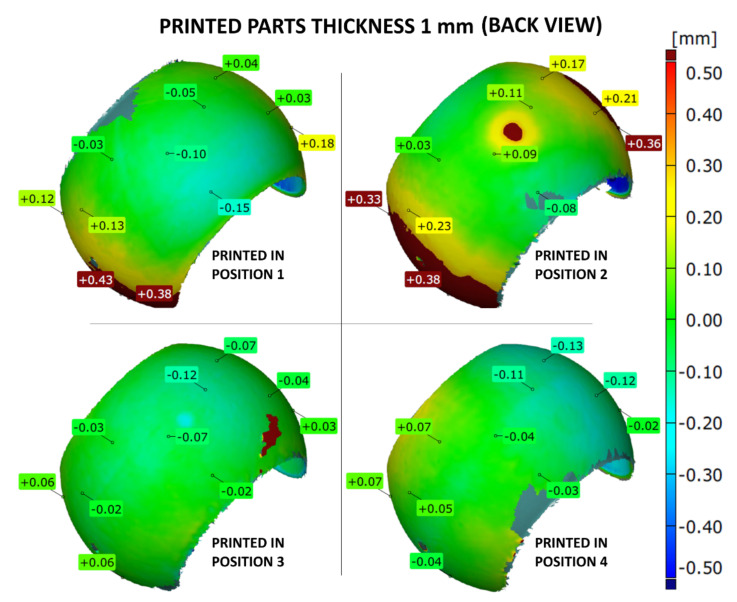
Geometry deviations of the 3D printed part with a thickness of 1 mm (back view).

**Figure 9:**
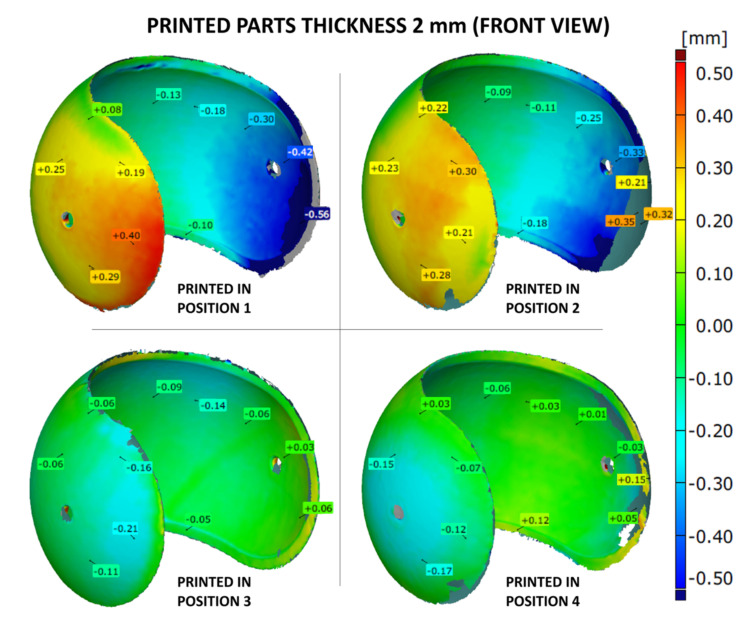
Geometry deviations of the 3D printed part with a thickness of 2 mm (front view).

**Figure 10:**
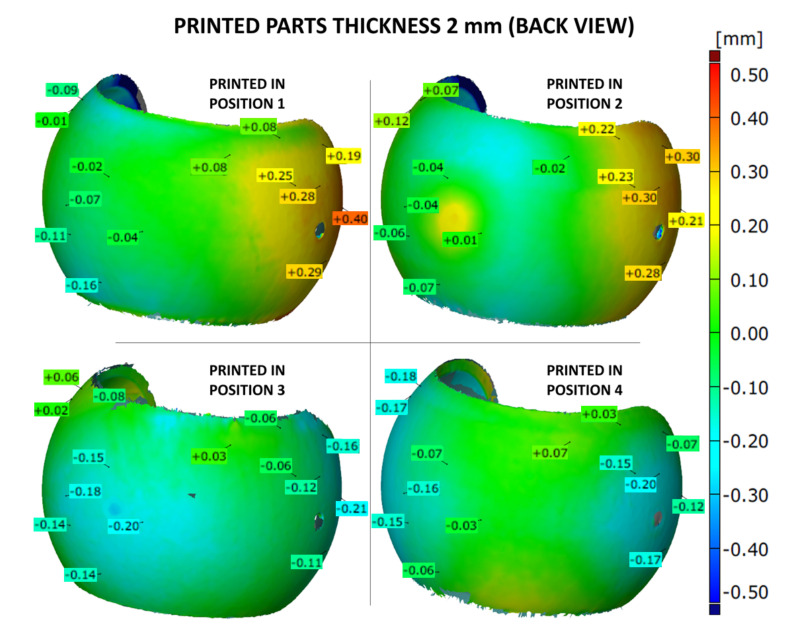
Geometry deviations of the 3D printed part with a thickness of 2 mm (back view).

**Figure 11:**
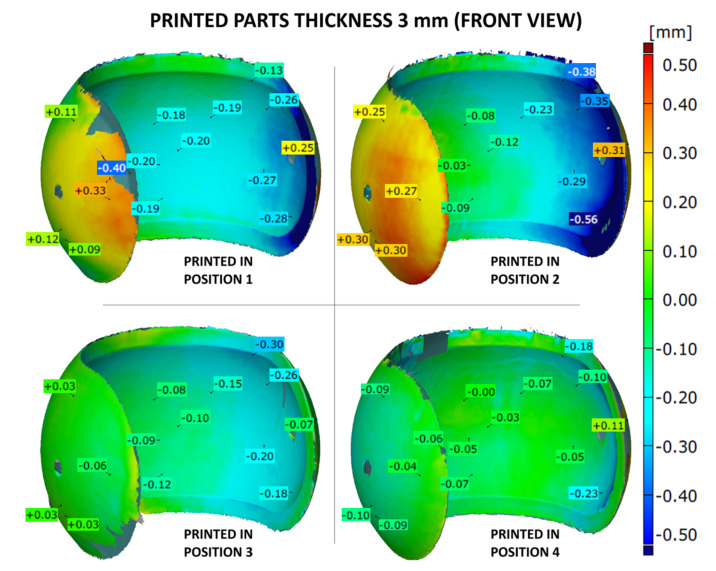
Geometry deviations of the 3D printed part with a thickness of 3 mm (front view).

**Figure 12:**
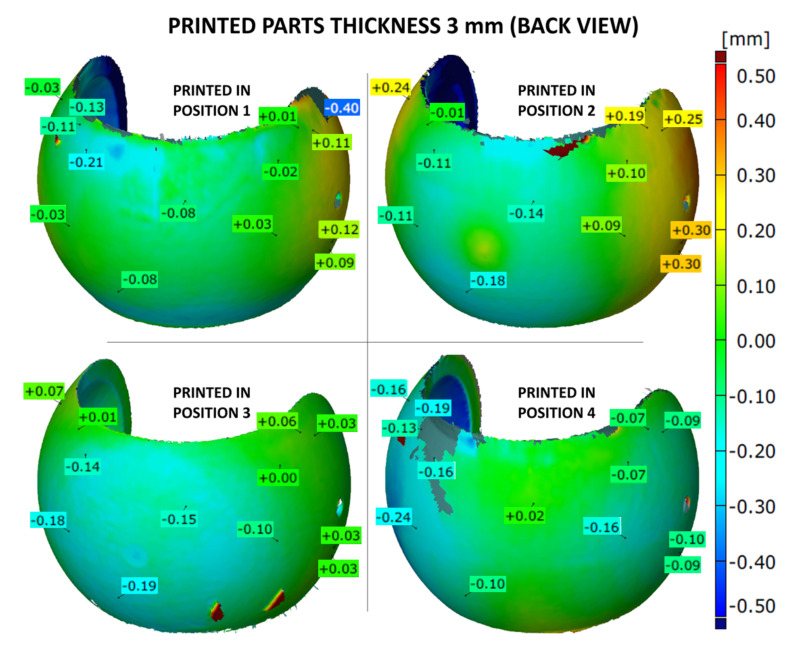
Geometry deviations of the 3D printed part with a thickness of 3 mm (back view).

The shape accuracy of the printed parts (with the wall thicknesses of 1, 2 or 3 mm) was verified by comparing the geometric deviations at selected points with the base .stl model. The results are given in Figure 7, Figure 8, Figure 9, Figure 10, Figure 11 and Figure 12. The distribution of deviations is presented in visual form using colors. Green color represents full dimensional model-to-part compatibility. Yellow up to deep red indicate positive deviation distribution, while light blue up to navy blue show maximum negative deviations.

The shape accuracy of a 1 mm thick sample printed in four different positions in comparison with the original model is shown in Figure 7 and Figure 8. The most apparent shape deviations occurred in print orientation no. 1 and 2, near the model’s free edges. Position 2 seems to be strongly unfavorable for 1 mm thick model as it is characterized by high dimensional deviations, both positive and negative, reaching up to +0.45 mm and −0.47 mm. Furthermore, print orientation no. 2 is the only one where significant deviations were also observed at the back side of the print (Figure 8), which in discussed position was nearly parallel to the XY plane.

The parts manufactured according to orientations 3 and 4 more precisely resembled the original .stl models. The best fit was provided by position 4, with deviation ranging from −0.16 mm up to +0.07 mm.

For 2 mm print thickness, the model-to-part deviation distributions were quite similar as in the case of 1 mm parts, for the respective building positions (Figure 9 and Figure 10). In case of print positions no. 1 and 2, the free edges of prints were characterized by positive deviations, suggesting a broadening of the print in that place. As it was revealed for thinnest parts, the 2 mm samples built in positions 3 and 4 have the lowest deviation rates. However, in these positions, some areas showed negative deviation. This was caused by the so-called capillarity effect, which affects large horizontal top surfaces. Finally, it seems that position 4 resulted again in overall highest shape accuracy, with very slight model-to-part differences.

The dimensional deviations for thickest, 3 mm, prints are presented in Figure 11 and Figure 12. The 3D maps are very similar to the ones obtained for thinner samples. The overall values of dimensional deviations seem to be slightly lower than for 1 or 2 mm prints. The capillarity effect is still present but less evident as well. Furthermore, the part produced according to position 4 revealed again the lowest dimensional deviations in the analyzed batch of 3 mm thick samples.

**Figure 13:**
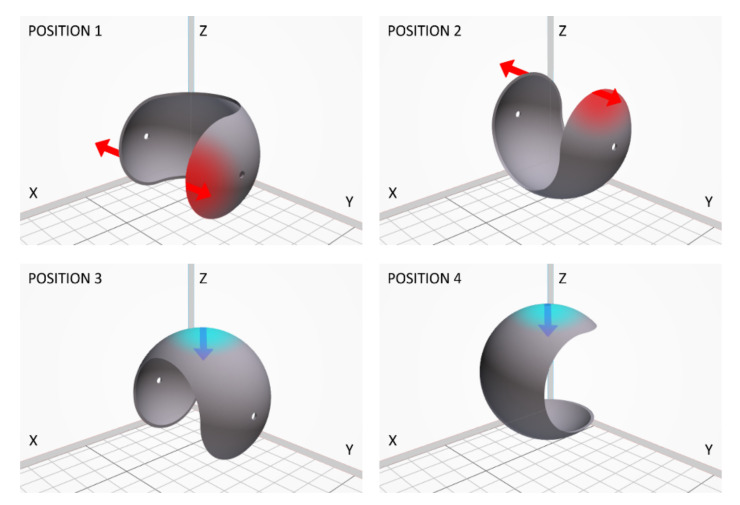
Graphical summary of the 3D scanning investigations for all print orientations.

The results obtained by 3D scanning showed that the increase in part thickness shows slightly beneficial effect for obtaining higher model-to-part accuracy in Multi Jet Fusion. However, the orientation in the printing chamber has an apparent impact on dimensional precision. The study showed that the preferred position is no. 4, regardless of the element thickness. The most important findings observed by 3D scanning are presented in Figure 13. For part build orientation no. 1 and 2, the parts’ open contours are deformed due to significant heating of the element itself, as well as loose (unbound) powder inside the sphere. This effect does not occur with parts printed in positions 3 and 4, because the bottom side of element is not heated enough to be deformed. The deformation could take place in the upper sides of prints due to higher temperatures, but it is blocked because of the closed contour. However, for printing positions no. 3 and 4 a slight collapse of the top surface occurs, which is characteristic for curved, thin elements exposed to non-uniform cooling. In such case, there is an uneven shrinkage of flat areas of prints prone to deformation. This effect is also favored by shrinkage and surface capillarity, quite typical for powder bed fusion technologies, and as a result, the consolidated areas tend to remain higher around the perimeter [37]. It should be noted that apart from changes in geometry, the orientation of printed parts in the chamber may also affect the microstructure, and thus, mechanical properties of prints [38,39].

### 3.2. Shape and Dimensional Analysis of Prints as a Function of Orientation in the Chamber

**Figure 14:**
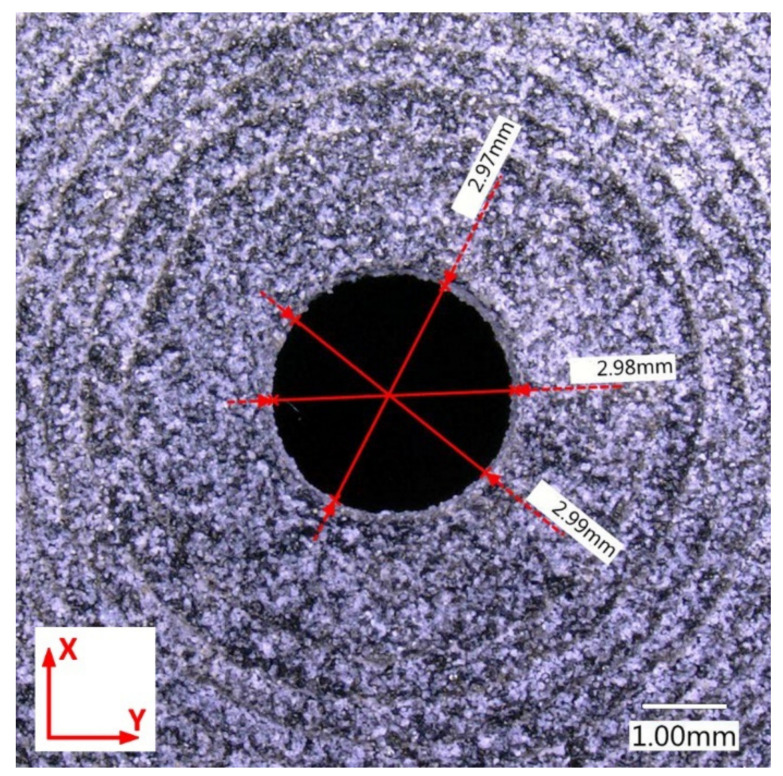
Measured hole diameter of part 4.3.

**Figure 15:**
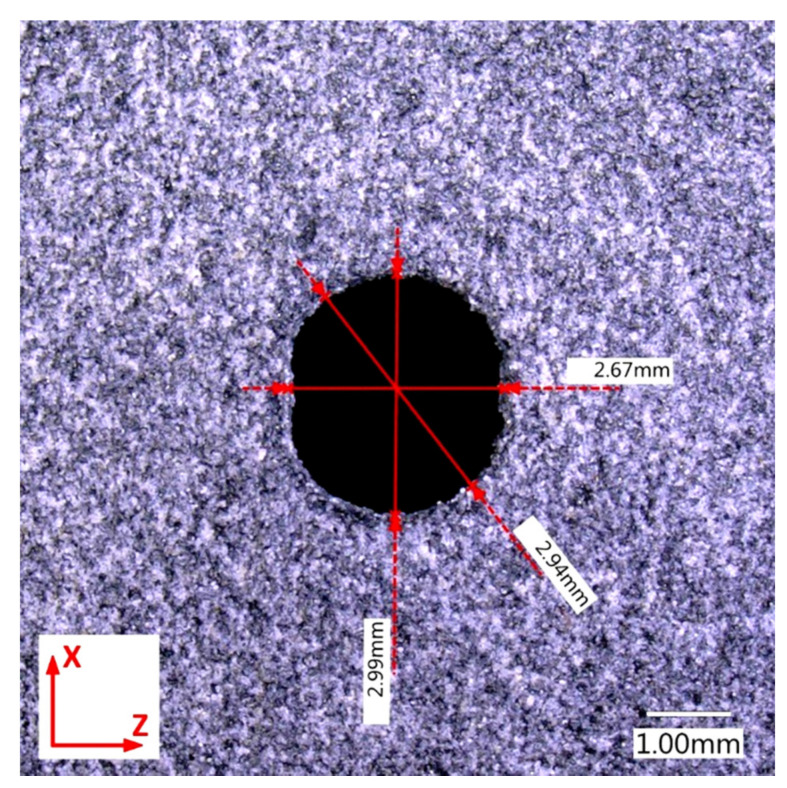
Measured hole diameter of part 1.3.

First, the diameter of holes was measured, which was set at 3 mm for all .stl models. The measurements showed that this value was consistent only for orientation 4 (Figure 14). For three remaining build directions, deviations from the circular to oval shape were clearly observed (see for example Figure 15, for orientation 1). The change, i.e., reduction, in diameter size (reduction) reached even 10% and this was similar for orientations 1–3. The described tendency was observed for all three analyzed print thicknesses. This is consistent with the recommendations of HP [40], suggesting to print holes along the X-Y plane in order to achieve highest resolution in such cases in MJF technology.

**Figure 16:**
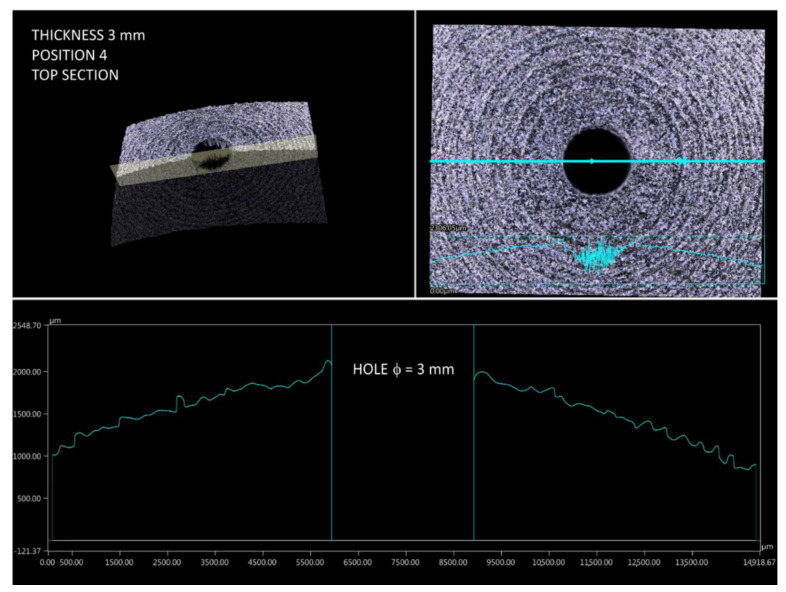
Surface profile of part 4.3 (top section).

Then, the curvature profiles of prints for different build orientations were analyzed (according to Figure 4). Measurements were made in two mutually perpendicular planes crossing the hole. Then, based on the profiles taken for all four print orientations it was possible to identify challenging fragments in terms of dimensions and shape. Considering the setting of samples during the investigations, an interesting phenomenon was observed for prints in position 4. The stair-stepping structure was clearly visible in the top section of part, near to the hole (Figure 16). This is characteristic for all layer-by-layer deposition technologies, to which MJF obviously belongs. In this analyzed case of stair-stepping, the structure in the upper section appeared up to an angle of about 15° (tangent to the circle), and then, up to an angle of about 20° it transformed into a transition region with increased roughness. Then, at an angle of 20°, the surface finish was basically impeccable.

**Figure 17:**
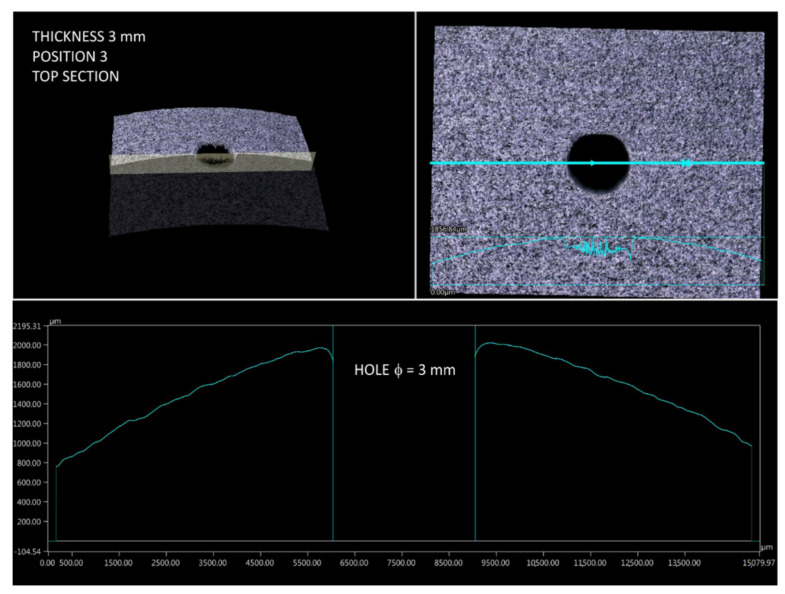
Surface profile of part 3.3 (top section).

The stair-stepping effect was not observed in any other build orientation for the specific prints’ parts intended for microscopic observations. All of them were printed parallel to the side walls of the build unit, and all of them showed similarly high surface quality (see example in Figure 17). This is in line with the results of studies by Cai et al., who showed that MJF gives better surface finish in the front and side surfaces (along X and Y build unit axes) compared to the top one (along Z axis) [38]. It should be emphasized that the size of the stair-stepping structure is influenced by many factors, including dimensions and volume of elements, degree of parts compaction in chamber, etc.

**Figure 18:**
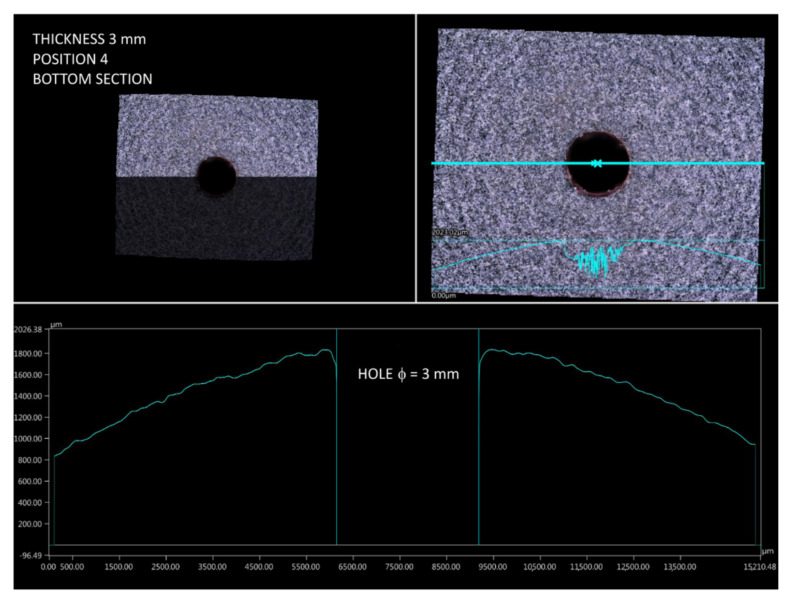
Surface profile of part 4.3 (bottom section).

One would expect a similarly defective area to appear in the bottom section of a print when built according to position 4. However, as a result of the more favorable heat distribution at this region, the curvature profile is much smoother and closer to being circular. In the analyzed case, there was no stair-stepping structure at all (Figure 18).

**Figure 19:**
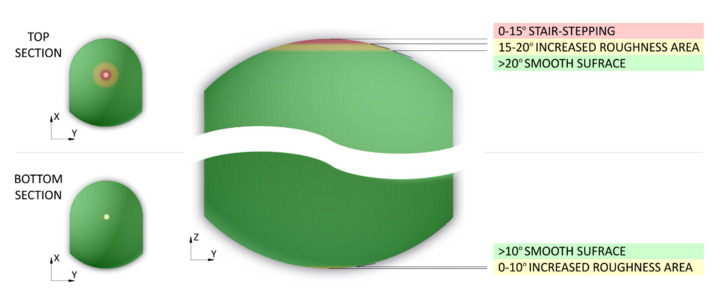
The occurrence potential for stair-stepping on the example of parts printed in orientation 4.

The results of the microscopic observations and measurements are summarized in Figure 19, showing the potential stair-stepping occurrence on the example of parts printed in orientation 4. The effect can be limited before starting the printing process by using a dedicated numerical simulation software that predicts the quality of the print surface in a selected print orientation [41]. Additionally, if necessary, it may be reduced in post-processing, e.g., by application of different coatings to the 3D printed parts [42].

To confirm the differences in surface quality in the three analyzed areas, roughness measurements were performed. It should be emphasized that the results of roughness measurements are closely related to usually post-processed MJF prints (for MJF abrasive blasting with glass balls is recommended). Therefore, the presented results should be interpreted qualitatively, not quantitatively. In Table 1, the top, middle and bottom sections of parts printed in orientation 4 were analyzed for each thickness (1, 2 and 3 mm). It is easy to notice that the difference in selected roughness parameters depends on the arrangement of printed parts in the build unit. The top section shows a more than two-fold increase in roughness compared to the middle section (the most favorable roughness parameters) and bottom section. Interestingly, along with the increase in wall thickness of printed parts, a slight decrease in all roughness parameters was observed (for 2 mm thick parts: Ra 3%, Rz 4%, Rt 6% and for 3 mm thick parts: Ra 6%, Rz 9%, Rt 16%; average values). This may be explained by the different heat distribution as a function of the thickness of the element.

### 3.3. Microstructural Analysis of Fractured Surfaces

The SEM studies allowed to observe and better understand the fused particulate nature of the print, depending on its thickness and build orientation.

**Figure 20:**
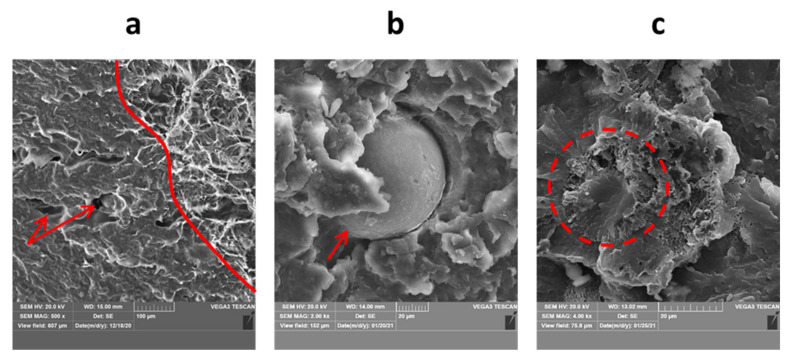
The most typical features observed in parts fabricated: (**a**) typical microstructure (part 1.3), (**b**) completely non-processed powder particle (part 3.2), (**c**) almost fully processed powder particle (part 4.2).

There are some general findings valid for all MJF specimens investigated in this work and they are discussed in the following paragraphs. The most typical fracture surface observed here for MJF prints is presented in Figure 20a, including both, ductile and brittle fracture surfaces. The prints are characterized by low porosity volume (usually mentioned to be maximally a few vol.%, studied, e.g., in [43]), and the pores were found to be rather randomly distributed and nondependent on the part thickness. Usually, there is no correlation between the porosity level and the specific regions of the fracture surface, apart from the interlayer porosity found in the case of orientation 4, which is later discussed in detail.

All fracture surfaces revealed intralayer porosity, voids and non-processed powders. They were all observed usually in low volume, which is also clarified later. So-called intralayer porosity is a structural defect that may be created in powder bed fusion processing mainly during the sintering stage of the powder particles as a result of poor fusing of powder particles, which produce later empty spaces in the print structure. Therefore, the intralayer pores may be of slightly different morphology and size, i.e., irregular or spherical with a size similar to one or several powder particles, but usually they are of ovoid shape (see Figure 20a, labeled with arrows). Such porosity is observed in different powder-based manufacturing processes [44], including additive manufacturing [45,46] and Multi Jet Fusion as well [47]. The selection of proper process parameters and reduction of print porosity is important for building parts with proper functional properties.

Single, non-processed, PA12 powder particles were observed in each print’s fracture surface. These are shown in Figure 20b,c, and include completely nonmelted and almost melted powder. This could be possibly due to the lack or nonhomogeneous injection of detailing agent, which could, in turn, locally affect the melting and fusing process in MJF. However, the number of such non-processed powders is still lower than in competitive Selective Laser Sintering, as presented in [32].

**Figure 21:**
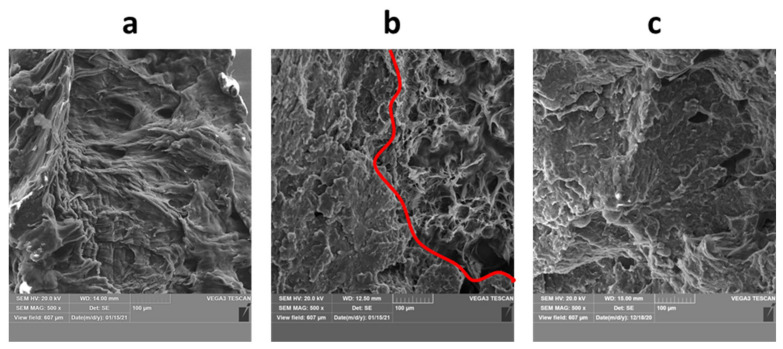
The fracture modes observed for prints with different thickness: (**a**) 1 mm (sample 1.1), (**b**) 2 mm (sample 1.2) and (**c**) 3 mm (sample 1.3).

Some specific phenomena, valid only for some specific samples, depending on their thickness or build orientation, were also observed and discussed below. All studied surfaces contain ductile and brittle fracture regions (Figure 21a–c), similarly as observed in [48]. However, the ratio of ductile to brittle area was found to be dependent mainly on the print thickness. The lower the wall thickness, the more intensive ductile deformation in the fracture surface was observed (Figure 21a), and the greater area was covered by the ductile fracture. Then, by increasing the wall thickness, the mixed, ductile, and brittle mode of fracture was observed (Figure 21b), while for the thickest prints the fracture was mainly brittle (Figure 21c). The mixed fracture mode was also observed by Rosso et al. [48] but instead was linked to specific regions in the print, i.e., more ductile in the upper part of fracture surface and more brittle in the lower parts. Furthermore, other powder bed fusion processes, like SLS, show rather more brittle behavior [49], which may suggest that MJF provides prints with higher toughness and capable to absorb more energy before final failure.

**Figure 22:**
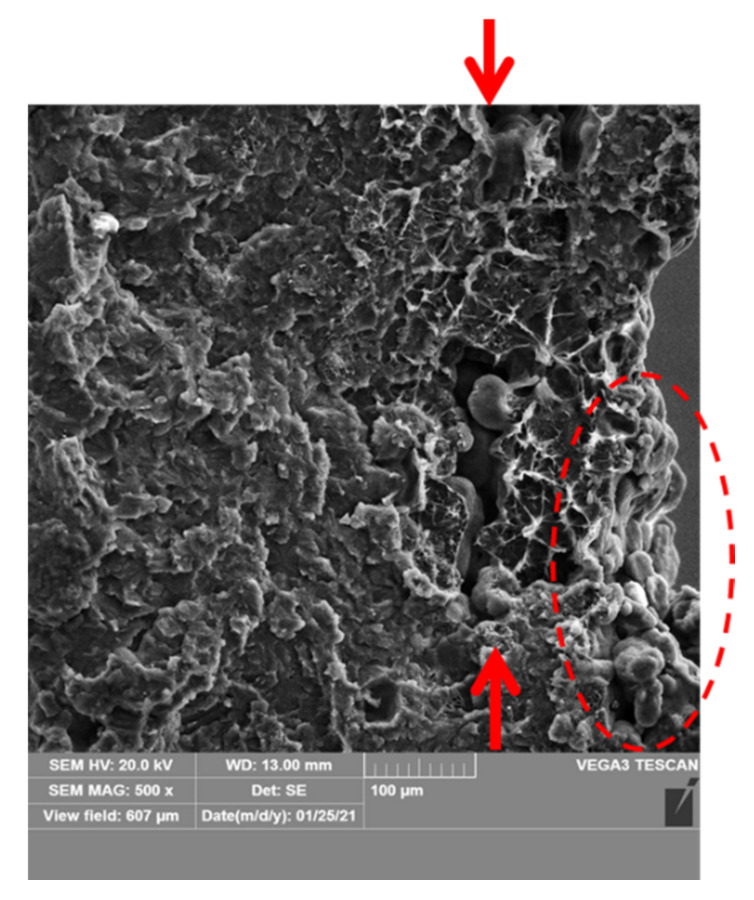
The voids occurring in the thinnest prints (1 mm), just close to the sample surface (sample 4.1).

Furthermore, the prints with the lowest wall thickness (1 mm) showed some big, longitudinal voids just close to the outer surface (labeled with an arrow in Figure 22), which suggest some issues with polymer consolidation and melt behavior [44]. It can be caused by insufficient energy input in that region, so the material is not fully melted. This, in turn, may be linked with some problems with distribution of fusing and/or detailing agents for such thin structures, as usually there are always non-processed powders on the surface of prints (as labeled by dashed line in Figure 22).

**Figure 23:**
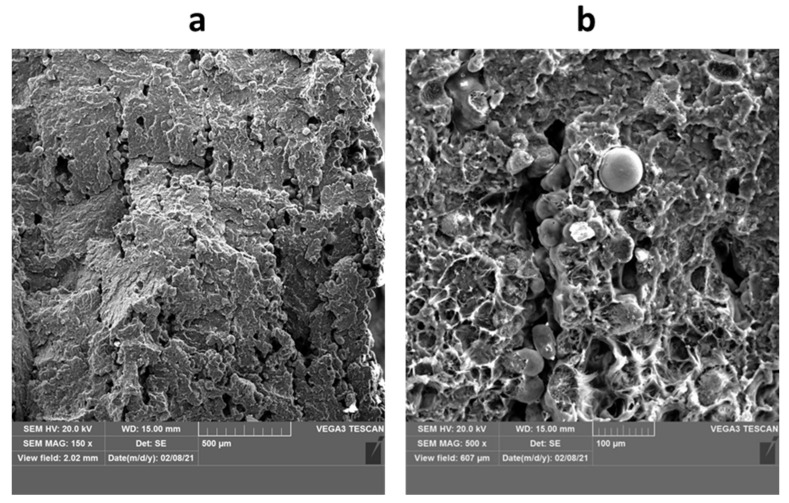
The interlayer porosity and unmelted tracks of powders observed for orientation 4, sample 4.3: (**a**) low magnification (150×) and (**b**) higher magnification (500×).

Finally, orientation no. 4 was found to be the worst one in terms of print’s structure quality, especially for the thickest parts. These samples were characterized by the highest (interlayer) porosity and the biggest number of non-processed powder particles in the fracture surface, which is shown in Figure 23. The interlayer porosity and unmelted tracks of powders may be a reason for the nonhomogeneous deposition of powder layers or too low heat input necessary to fully process the powder layer. These issues may be also related to the powder characteristics, morphology and uniformity (as reported for direct energy deposition additive manufacturing [50]), especially if we consider that in MJF the conventional powder mix is 80:20 (i.e., only 20 wt.% of new powder in the build unit). The influence of powders on the print structure was reported for other additive manufacturing. Furthermore, a similar effect was observed for fracture surfaces in both bottom and top regions of the print manufactured in orientation 4. This suggests that this issue can be met at any build height and there is no direct correlation between the part height (along the Z axis) and porosity. This was observed previously for PA11 prints fabricated by Multi Jet Fusion technology [51].

## 4. Conclusions

This study investigated geometry, dimensional accuracy, and structure of thin-walled spherical parts, which were successfully additively manufactured by Multi Jet Fusion. The PA12 prints were produced under the balanced mode and then, the influence of the part thickness (1, 2 or 3 mm) and four various build orientation were considered here.

The studies showed that the thicker parts are characterized by slightly higher dimensional and geometrical accuracy but, in fact, the print’s thickness is not a key factor here. Significantly more important is the part build orientation. The 3D scanning and macroscopic investigations showed that the worst part accuracy is obtained for orientation no. 1 and 2, i.e., with the open part’s contour facing the top of print chamber. On the contrary, the prints produced in orientation no. 4 are of highest model-to-print accuracy, with only some local differences in the dimensions or shape, like so-called capillarity or stair-stepping, caused by the MJF process characteristics itself. For prints produced according to orientation 4 it was also noticed that the roughness of the prints is the highest in the top part region and slightly decreases with the increase in parts’ thickness.

The microstructural analysis of fracture surfaces revealed that the most typical fracture surface observed in MJF prints includes both, ductile and brittle regions. In general, the prints are characterized by low porosity with pores rather randomly distributed in the print’s cross-section. Some single non-processed powders are usually embedded in the print structure as well. Furthermore, non-dependently on the print orientation, in the thin parts (1 mm) some issues with powder processing were observed close to the outer surface, which caused longitudinal voids, parallel to the print surface. This is still quite far from the thickness limit, recommended to be 0.6 mm for that technology. Finally, it should be noted that, contrary to geometrical accuracy, the printing orientation no. 4 was the worst one in terms of print’s structure quality because of the great (interlayer) porosity and the extremely high number of non-processed powder particles, especially for the thickest parts. This suggests that the print structure influencing e.g., mechanical properties, does not go hand-in-hand with printing accuracy and surface quality.

## Figures and Tables

**Table 1:** Surface roughness of parts printed in orientation 4.

Part Thickness	Part Section	Ra	Rz	Rt
1 mm	top section	20.1	93.6	126.2
	middle section	8.1	43.2	56.7
	bottom section	9.8	52.2	68.1
2 mm	top section	19.7	89.9	118.6
	middle section	7.9	41.9	53.9
	bottom section	9.4	51.2	63.3
3 mm	top section	19.1	85.2	104.7
	middle section	7.6	39.7	47.6
	bottom section	9.3	48	57.9

## Data Availability

Data sharing is not applicable to this article.
